# Microfluidic Impedimetric Cell Regeneration Assay to Monitor the Enhanced Cytotoxic Effect of Nanomaterial Perfusion

**DOI:** 10.3390/bios5040736

**Published:** 2015-11-27

**Authors:** Mario Rothbauer, Irene Praisler, Dominic Docter, Roland H. Stauber, Peter Ertl

**Affiliations:** 1 BioSensor Technologies, AIT Austrian Institute of Technology GmbH, 1190 Vienna, Austria; E-Mails: mario.rothbauer@gmail.com (M.R.); ipraisler@gmail.com (I.P.); 2 Molecular and Cellular Oncology, ENT/University Medical Center Mainz, 55116 Mainz, Germany; E-Mails: docter@uni-mainz.de (D.D.); rstauber@uni-mainz.de (R.H.S.)

**Keywords:** lab-on-a-chip, cell chip, impedance, biosensor, nanoparticle, lung cancer, nanotoxicology

## Abstract

In the last decade, the application of nanomaterials (NMs) in technical products and biomedicine has become a rapidly increasing market trend. As the safety and efficacy of NMs are of utmost importance, new methods are needed to study the dynamic interactions of NMs at the nano-biointerface. However, evaluation of NMs based on standard and static cell culture end-point detection methods does not provide information on the dynamics of living biological systems, which is crucial for the understanding of physiological responses. To bridge this technological gap, we here present a microfluidic cell culture system containing embedded impedance microsensors to continuously and non-invasively monitor the effects of NMs on adherent cells under varying flow conditions. As a model, the impact of silica NMs on the vitality and regenerative capacity of human lung cells after acute and chronic exposure scenarios was studied over an 18-h period following a four-hour NM treatment. Results of the study demonstrated that the developed system is applicable to reliably analyze the consequences of dynamic NM exposure to physiological cell barriers in both nanotoxicology and nanomedicine.

## 1. Introduction

Nanomedicine, defined as the application of nanotechnology to healthcare, is an emerging field that promises to facilitate biomedical research and significantly improve medical care [1]. Nanomedicine consists of nanodiagnostics, targeted drug delivery and regenerative medicine and is expected to greatly impact the prevention, reliable diagnosis and treatment of disease [2]. Although nanomedicine is a relatively new field of research, the technology on which it is founded first appeared over a decade ago. For instance, nanoscale technologies, such as colloidal gold, quantum dot semiconductor crystals and iron oxide crystals capable of targeting different cells and extracellular components in the body, are already used to deliver drugs, genetic material and diagnostic agents [3,4,5]. The use of magnetic nanoparticles to separate, isolate and detect cells or molecules, such as proteins, peptides and DNA, even dates back to the early 1990s [6]. More recently, however, intra-venous (i.v.) injection of nanoparticles is used in the specific delivery of chemotherapeutic agents and diagnostic agents, as well as in the elimination of cancer cells [7]. However, one should not neglect that these developments will also lead to an increasing exposure of humans and the environment to nanomaterials. Once inside cells, NPs may cause adverse effects and even permanent cell damage [8,9,10]. As potential mechanisms, oxidative stress, inflammation, genetic instability and the inhibition of proper cell division have been described, which, depending on the (patho)physiological context, may contribute to cell death [11,12,13,14]. Thus, the discussion about nanosafety aspects and regulations is certainly important and still ongoing [15,16,17,18].

As the safety and efficacy of nanomaterials (NMs) are of utmost importance, new methods are needed to study the dynamic interactions at the nano-biointerface. Risk assessment of nanomaterials is becoming increasingly important, because of the rising availability of nano-based products on the market. Since the nanocarrier itself is based on its perceived low toxicity (e.g., iron oxide up to 5 mg/mL), the cytotoxic potential of novel nanomaterials is a key parameter that must be thoroughly analyzed during the developmental stage using standard *in vitro* methods. A major drawback of existing cell-based nanotoxicological assays, however, is that they are conducted using static cell culture conditions employing endpoint detection methods [19]. It is important to note that besides physical and chemical properties of the nanomaterial, also external factors, such as exposure scenarios, including perfusion, influence the cell-particle interaction and therefore modulate toxicity. For instance, it was recently demonstrated that the toxicity of silver NPs increased under perfusion [20], while the added shear stress alone had only minor biological effects [21], indicating higher NP uptake rates in dynamic settings. In turn, decreased toxicity was found using fibroblasts that were perfused with quantum dots when compared to static exposure conditions, thus pointing to increased cell stress and lower viability in the presence of particle sedimentation [22].

To overcome these experimental inconsistences and to address existing limitations of end-point detection methods, including low reproducibility, reliability and accuracy, we have developed a microfluidic regeneration assay to monitor the cytotoxic potential of nanomaterials on *in vitro* cell cultures. Non-invasive monitoring of cell responses is accomplished using embedded impedance microsensors to readily identify morphological changes in the presence of nanomaterials. In a previous work, we have already applied cellular electric impedance sensing to study the mitigating effects of nanoparticle corona formation on cytotoxicity, thus highlighting the importance of media compositions on nanomaterial-cell interactions [23,24,25,26]. Cellular electric impedance sensing is considered a powerful cell analysis technique and has already been successfully applied to cancer research, determining the effects of novel drug candidates, for instance, on viability [27], proliferation [28] and morphology [29], as well as the identification of cellular activity between normal *vs.* malignant cells and highly- *vs.* poorly-metastatic cancer cells [30,31,32].

In the present work, we have developed a lab-on-a-chip to assess the regenerative capacity of human H441 lung adenocarcinoma cells following a four-hour administration of silica nanoparticles under varying flow conditions. Pulsatile blood flow *in vivo* is known to generate physical forces on cells. Therefore, the investigation of shear stress and stretching is important as parameters that can influence nanoparticle uptake and, thus, nanotoxicology. While cyclic stretching reduces the uptake rate of silica nanoparticles, microfluidics cell cultures have been shown to either increase or decrease the uptake of semiconductor quantum dots (QD) and SiO_2_ particles in the presence of elevated flow velocities [33,34]. To study the influence of flow velocity on the cytotoxicity of nanomaterials, we have employed a well-established nanotoxicological lung model based on toxic silica nanoparticles (AmSil30) and H441 cells. Using this nanotoxicological model, we have previously shown that serum supplements can alter the bioactivity of nanomaterials, thus modulating the cytotoxicity of silica nanoparticles. [24,35] Based on these results, nanoparticle exposures are performed using serum-free media in our microfluidic regeneration assay to study the effect of shear force on nanoparticle toxicity.

## 2. Experimental Section

### 2.1. Cell Culture

The hyperdiploid epithelial H441 cell line (lung papillary adenocarcinoma, H441 ATCC^®^ CRM-HTB-174™, ATCC) was cultivated in RPMI-1640 (Gibco 11875-093), 10% fetal calf serum (FCS; Gibco 16000-044) and 1% penicillin/Streptomycin (Gibco 15140-122) at 37 °C and 5% CO_2_.

### 2.2. Off-Chip Cytotoxicity Assays Using Standard Cell Culture Conditions

Standard cytotoxicity assays commenced with H441 cells seeded at a concentration of 80,000 cells/well and cultivated under standard conditions in standard 96-well plates (Corning^®^) until a cell surface coverage of 50% and 100% was reached. After this initial propagation period, medium was aspirated, replaced with serum-free medium, and cells were incubated for 60 min prior to nanoparticle administration. Next, the medium was changed to serum-free medium containing AmSil30 (NexSil20; NYACOL Nano Technologies, Inc., Ashland, MA, USA) for 4 h. The regeneration phase was initiated by aspirating the AmSil30-containing medium with serum-containing medium for an additional 20 h. Cell viability and metabolic activity was tested using tetramethylrhodamine ethyl ester perchlorate (TMRE; 87917, Sigma Aldrich, Vienna, Austria) and standard MTT assays, respectively.

The determination of cell viability was based on fluorescent membrane potential stain TMRE added to the well plates at a concentration of 25 nM in complete culture media and incubated for a period of 20 min at 37 °C in the absence of light. Further, a LIVE/DEAD^®^ Viability/Cytotoxicity Assay Kit (Life Technologies, Fisher Scientific GmbH, Vienna, Austria) staining solution in PBS (1 µL Component A and B mL^−1^) was prepared according to the manufacturer’s instructions. The culture medium was removed by rinsing with DPBS; 500 μL of the staining solution was added to each microwell and incubated for 10 min at room temperature. For both assays, fluorescence images were taken using a TE2000-S inverted fluorescence microscope (Nikon) equipped with a DS-Qi1Mc digital camera. All fluorescence images were recorded using a TRITC filter block (excitation at 540 nm, emission at 605 nm; Nikon) and processed using the manufacturer’s NIS-elements software (Nikon). For assessment of cell viability within the microfluidic chip LIVE/DEAD^®^ Viability/Cytotoxicity Assay Kit (Life Technologies, Vienna, Austria), staining solution was prepared according to the manufacturer’s instructions. The culture medium was removed by rinsing the microfluidic chip with DPBS; 200 μL of the staining solution was injected into the microchannel and incubated for 10 min at room temperature.

Evaluation of the metabolic activity was conducted using MTT (3-(4,5-dimethylthiazol-2-yl)-2,5-diphenyltetrazolium bromide, Sigma Aldrich, Vienna, Austria) added to the well plates at a concentration of 650 μg/mL in culture media. After 1 h at 37 °C, the MTT/medium mix was exchanged with 200 μL DMSO/well (A3672,0250; AppliChem GmbH, Darmstadt, Germany). The well plate was then wrapped in aluminum foil for light protection and mechanically agitated for 30 min at RT on a shaker. The well plates were analyzed via an EnSpire^®^ Multimode plate reader (excitation at 570 nm, absorption at 690 nm; PerkinElmer, Vienna, Austria).

### 2.3. Lab-on-a-Chip Fabrication

A detailed description of our 3 × 3 cm hybrid microdevices fabrication methods, including standard clean room processes and soft lithography techniques, can be found elsewhere [36,37]. The biochip was comprised of a glass-polydimethylsiloxane (PDMS)-glass sandwich architecture containing four cell cultivation chambers each consisting of individual addressable interdigitated electrode structures (IDEs) as impedance sensors. Excess ports for the fluidic inlets and outlets were drilled at the glass top of the chip and connected via tubing to external syringe pumps. The middle layer was composed of PDMS (SYLGARD^®^184 Silicone Elastomer Kit, Dow Corning GmbH, Wiesbaden, Germany) and defined the size and volume of the cell culture chambers. The bottom of the chip was made of glass (Schott, Borofloat^®^) containing 50 nm-thick sputtered gold IDEs (20-µm spacing and 20-µm finger width), leads and contact pads. The IDEs were connected using spring-loaded PCB connectors via wires to a VMP3/P-01potentiostat (BioLogic), and data were recorded using the manufacturer’s EC Lab software (v10.34).

### 2.4. Device Preparation and on-Chip Impedimetric Regeneration Assay

Device preparation for cell culture handling included rinsing with 70% ethanol at a flow rate of 40 µL/min for a minimum of 40 min. The system was then heated to 37 °C; complete cell culture medium was introduced for more than 1 h prior to experimentation. Bubble-free cell inoculation was performed through a four-way valve typically using concentrations of approximately 400,000 cells/mL seeded into the cell culture chambers. Following a 30-min cell attachment period under static conditions, medium perfusion was activated using the syringe pump at a flow rate of 4 µL/min. Continuous and uninterrupted medium was supplied for an additional 2–3 days until the creation of confluent cell layers was visually confirmed. Nanoparticle administration was performed similar to the protocol used in off-chip cytotoxicity assays in the absence or presence of increasing flow rates (e.g., 4 µL/min or 40 µL/min). Following a 4-h incubation period, the cellular regeneration phase was initiated by replacing the AmSil30-containing medium with serum-containing medium for an additional 20 h.

## 3. Results and Discussion

### 3.1. Characterization of the on-Chip Impedance Biosensors

**Figure 1:**
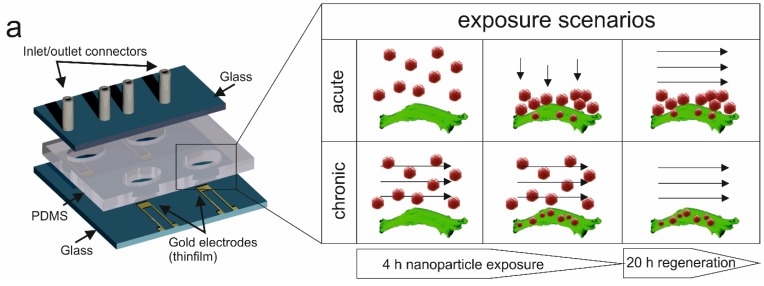
Schematic illustration of the lab-on-a-chip system for continuous and non-invasive monitoring of the cytotoxic potential of dynamic silicon nanoparticle administration. During the presented cell-based microfluidic assay, acute (static), as well as perfused (chronic) nanoparticle exposure scenarios can be simulated, and cancer cell regeneration is tested using impedance gold electrodes.

The key feature of the presented lab-on-a-chip system is the combination of multiplexed impedance biosensors with microfluidics to continuously and non-invasively monitor dynamic cell-nanoparticle interactions. Figure 1 shows the schematic layout of the developed lab-on-a-chip consisting of four cell chambers each containing an embedded impedance microsensor and selected exposure scenarios.

**Figure 2:**
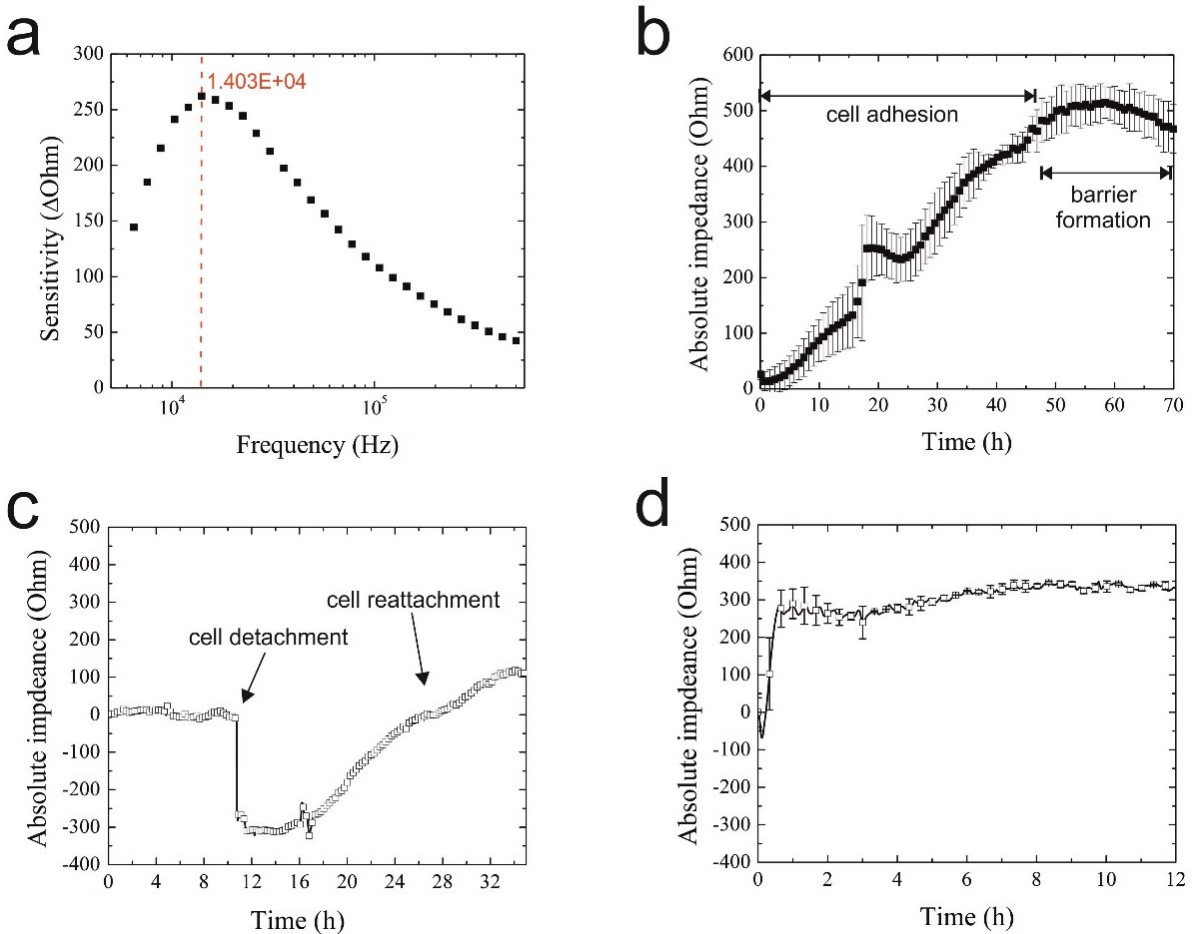
(**a**) Representative graph of interdigitated electrode structures (IDEs) sensitivity towards H441 epithelial cell monolayers in the range of 1 kHz–500 kHz with the highest signal change indicated in red; (**b**) typical on-chip adhesion curve of H441 cells (*n* = 3) on 20 µm × 20 µm IDEs over a period of 70 h; (**c**) representative graph of IDE response during H441 detachment and reattachment at a frequency of 14 kHz; (**d**) impedance time trace of H441 cell regeneration and proliferation 4 h post-starvation.

Since cell type and cell culture origin are known to affect the sensitivity and reproducibility of impedimetric biosensors, it is first necessary to assess biosensor performance using the respective cell culture model under investigation [38,39]. Initial impedance optimization included frequency analysis ranging from 1 kHz–500 kHz to determine the highest sensor sensitivity to H441 lung papillary adenocarcinoma cell lines. In Figure 2a, a representative sensitivity graph is shown exhibiting the highest signal change of 260 Ohms at 14 kHz using the 20-µm IDE sensors (finger-to-spacing ratio of 1:1). Sensor sensitivity is defined as the maximum signal change in the absence and presence of a fully-covered sensor surface with epithelial cells. To reduce the amount of generated data over a 2–3-day cell culture period, single frequency impedance measurements at 14 kHz were used for all subsequent on-chip experiments. To further demonstrate the reproducibility of the on-chip IDE impedance biosensors, cell adhesion curves are recorded in quintuplicates using different biochips and sensors. Results in Figure 2b show an overall signal variation of <10% between the different cell culture chambers at the time of barrier formation (e.g., max. 8.6% deviation from the mean value at t = 70 h), thus demonstrating the reproducibility of the microfluidic cell culture handling procedure. The observed impedance time traces point to similar cell adhesion, spreading and proliferation rates that lead to the establishment of a confluent monolayer and epithelial barrier formation 50 h post-cell seeding, as indicated by the impedance signal plateau.

Next, bioassay performance was investigated by studying sensor responses to detachment and re-attachment of cells. In a series of experiments, H441 cells were cultivated over 50 h until the formation of a tight epithelial barrier, while cell injury was simulated by mechanical cell detachment. The result of the assay performance analysis is shown in Figure 2c, where the initial impedance drop of 300 Ohm and signal recovery indicate rapid cell detachment followed by a 14-h regeneration phase and cell proliferation. Final bioassay characterization involved the assessment of the influence of medium composition on cell behavior, since nanoparticle administration is performed under serum-free conditions to avoid protein corona formation known to mitigate nanoparticle toxicity [24,35]. Impedance time traces shown in Figure 2d feature an immediate impedance trop indicating that H441 cells rapidly react to serum depletion (starvation). However, following the addition of 10% FCS, cells fully regenerated within 1 h post-starvation and continued to proliferate, as indicated by the rising impedance values for the subsequent 13 h, thus pointing to the feasibility of using cellular regeneration dynamics as an indicator of cell viability in nanotoxicological studies.

### 3.2. Toxicological Characterization of AmSil30 Nanoparticles

**Figure 3:**
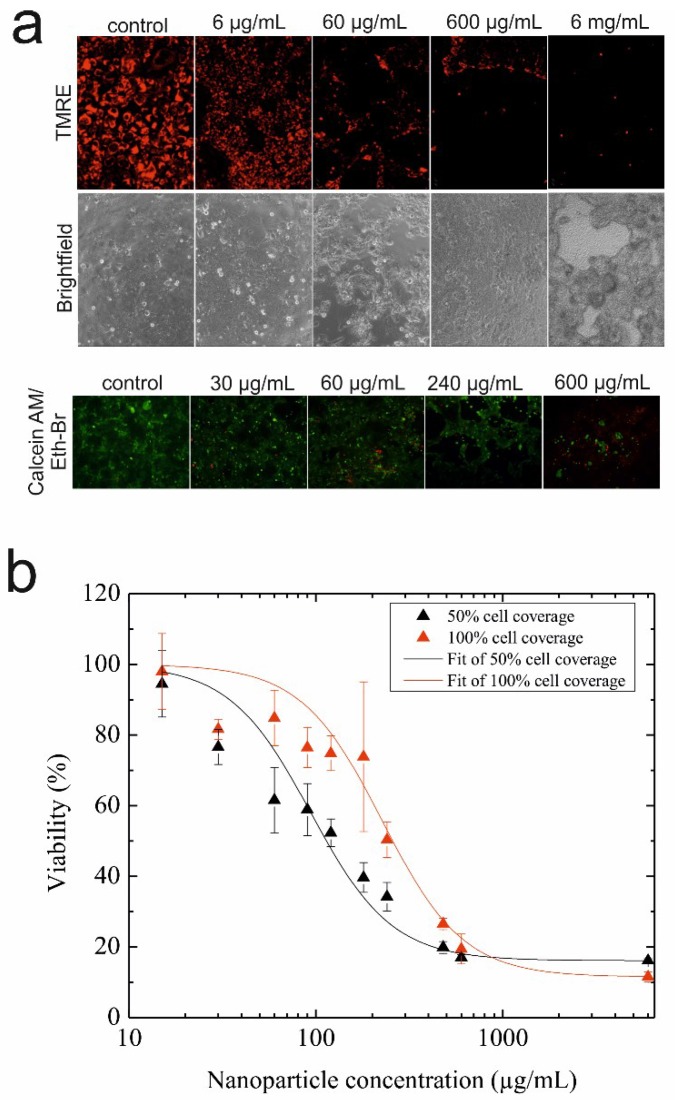
(**a**) Fluorescence and bright-field images of H441 cells stained with tetramethylrhodamine ethyl ester perchlorate (TMRE) mitochondrial membrane potential dye (live cells: red; top panel) and the live/dead cytotoxicity assay kit (live cells: green; dead cells: red; bottom panel) post-nanoparticle administration and regeneration. (**b**) Cytotoxicity of silica nanoparticles (AmSil30) towards 50% and 100% confluent H441 epithelial cell layers. The data presented are derived from the metabolic MTT assay (*n* = 3) and expressed as % mean value ± % standard deviation.

Before conducting on-chip microfluidic regeneration assays, the cell viability and metabolic activity of epithelial H441 cells were investigated using standard static cell cultures to identify sub-toxic, as well as EC_50_ values for AmSil30 silica nanoparticles. Results of this nanotoxicological study using TMRE mitochondrial membrane potential dye to indicate cell viability are shown in Figure 3a and revealed that AmSil30 nanoparticle concentrations between 600 µg/mL and 6 mg/mL are highly toxic for H441 cells, while 60 µg/mL showed reduced and 6 µg/mL almost no cytotoxicity effects. Similar results were obtained using a live/dead cytotoxicity assay kit, as shown in Figure 3a (bottom panel); however, for 60 µg/mL of silica nanoparticles, which was previously reported to have no significant effect on H441 cell viability [40], the two viability assays distinctly differed and showed that cytotoxic potential is clearly dependent on cell surface coverage. Therefore, to further investigate the effects of increasing silica nanoparticle concentrations on H441 epithelial cell cultures at different cell surface coverages, quantitative analysis of AmSil30 cytotoxicity was performed using MTT assays in subsequent experiments. Results of the viability study are shown in Figure 3b demonstrating a distinct dose-time response in the presence of different cell culture densities. While an EC_50_ value of 120 µg/mL was calculated for 50% confluence, a significant EC_50_ shift to 240 µg/mL was observed using a fully-confluent (100%) cell layer. These results indicate that islands of proliferating cells, as well as non-confluent cell layers react more sensitively to cytotoxic nanomaterials than intact epithelial cell monolayers that resemble an epithelial lung barrier.

### 3.3. Comparison of Acute and Chronic NP Administration Scenarios on H441 Tumor Regeneration

**Figure 4:**
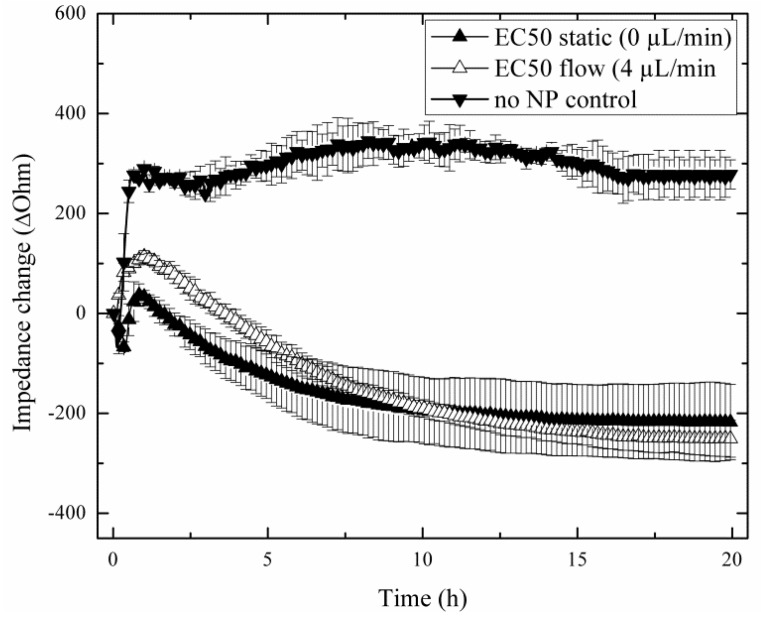
Impact of acute (static, *n* = 4; ▲) and chronic (flow, *n* = 3; ∆) silica nanoparticle (AmSil30) administration scenarios on H441 tumor regeneration over a 20-h regeneration phase with untreated cells (▼) as the control group (*n* = 3).

In a comparative study, two nanoparticle treatments are investigated that simulate either acute or chronic exposure scenarios to reflect real-world situations of the lung. In subsequent on-chip experiments, tight cellular barriers were first established within the cell chambers, and the influence of acute to chronic nanoparticle exposures on the capability of H441 tumor cells to regenerate are followed after a 4-h treatment period with the EC_50_ concentration of AmSil30 nanoparticles (240 µg/mL). Figure 4 shows three replicate impedance time traces of H441 tumor cell regeneration behavior over a period of 20 h using untreated, acute and chronically-exposed cell cultures (see also Figure 1). Results show an immediate impedance drop to −217.4 ± 75.98 Ohm in the presence of the acute (static) scenario and −250.7 ± 36.98 Ohm for the chronic (4 µL/min flow) exposure scenario, respectively. However, while in control experiments (no nanoparticles), H441 cells readily re-attached, leading to an impedance increase of +274.87 ± 29.17 Ohm; regeneration behavior varied distinctly in the presence of acute and chronic exposure scenarios. The observed continuous impedance signal loss after administration of nanoparticles points to progressive cell damage and cell death over the remaining 20 h cultivation period. These findings correspond well to the MTT results, which showed severe cytotoxic effect on H441 cells during 4 h of nanoparticle exposure. Although, similar in the overall shape, impedance time traces following acute and chronic exposure can be readily distinguished, thus indicating that nanoparticle administration influences regeneration behavior. Most notably, standard deviations of impedance traces and signal variance between experiments decreased in the presence of chronic (perfused) particle exposure, thus improving the reproducibility of the impedimetric microfluidic regeneration assay.

### 3.4. Impact of Increasing Flow Rates on Tumor Regeneration during NP Administration

**Figure 5:**
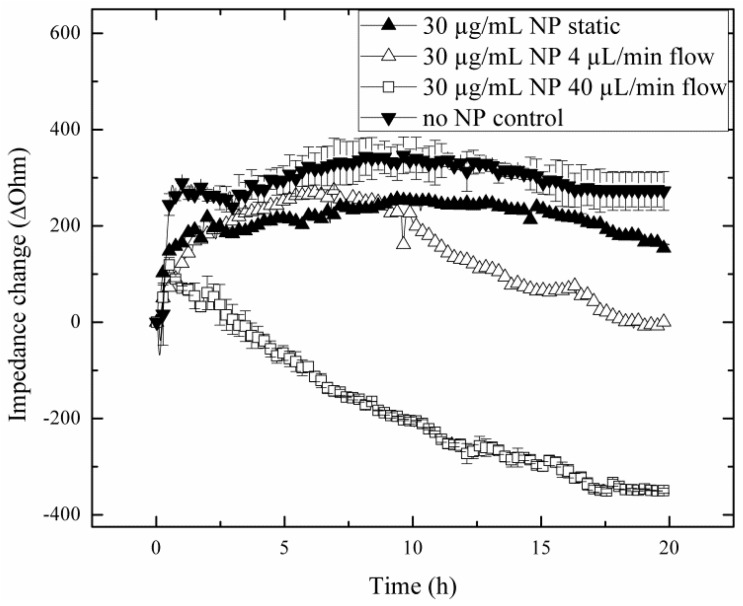
Impact of flow rate increase from 4 µL/min (*n* = 1; ∆) to 40 µL/min (*n* = 3; □) during the chronic silica nanoparticle (AmSil30) administration scenario at sub-toxic particle concentrations on H441 tumor regeneration over a 20-h regeneration phase with untreated cells (*n* = 3; ▼) and static exposure (*n* = 1; ▲) as control groups.

Since flow rates are known to influence nanoparticle uptake rates and toxicity, the impact of increasing flow rates on the capacity of lung cells to regenerate is investigated in subsequent experiments. Results of the nanotoxicological study are shown in Figure 5, where cellular regeneration in the absence and presence of sub-toxic concentration of silica nanoparticles (30 µg/mL) is monitored following exposure at 4 µL/min and 40 µL/min flow rates. Obtained impedance time traces of H441 lung cell regeneration are remarkably different between the various exposure scenarios, thus highlighting the importance of assessing cellular dynamic behavior. For instance, while similar regeneration behavior was found in the absence of flow (static exposure) and control experiments (no particle treatment), nanoparticle exposure under flow conditions resulted in decreased regeneration capacity, therefore pointing to increased toxicity. Interestingly, although the untreated control and the static exposure experiment revealed complete regeneration capacity already after 3 h, the lower impedance values and plateau level obtained in the presence of sub-toxic silica nanoparticles may indicate a loss in viability. In contrast to these results, administration of AmSil30 nanoparticles at an increased flow rate of 40 µL/min led to an immediate impedance drop to −355.26 ± 7.37 Ohm, indicating severe cell damage and death. Impedance time traces in the presence of 30 µg/mL silica nanoparticle look similar to those obtained under static treatment of H441 cell cultures using concentrations of 240 µg/mL or the EC_50_ level, thus indicating increased nanoparticle uptake during the 4-h exposure time. To clarify these results, H441 cells were subjected to a 40-µL/min flow rate for 4 h without the addition of silica nanoparticles. As seen in Figure S1, neither downward impedance drops nor morphological changes were observable for the regeneration of H441 cells exposed to 40 µL/min flow (0.0025 dyn/cm^2^) in the absence of nanoparticles. The most striking cellular regeneration behavior, however, is found following the exposure of a sub-toxic concentration of silica nanoparticles at a 4 µL/min flow rate. While an initial impedance increases of approximately 30 Ohm/h point to active regeneration behavior within the first 10 h, H441 lung cells underwent a period of diminished capacity to form a fully-regenerated tight epithelial cell barrier in the remaining 10 h.

## 4. Conclusions

Besides the physical and chemical properties of nanomaterials, also external factors, such as perfusion, influence the cell-particle interaction and, consequently, the intracellular uptake. It has been shown that results based on conventional static significantly differ from perfused cell culture systems. For instance, static conditions drastically affect the outcome of dosage optimization studies and bioactivity assays due to the gravitational settling of NPs [26]. To study the influence of flow velocity on cell-based assays, microfluidic devices have become an attractive tool to provide defined and reproducible stimulation scenarios that allow the reliable investigation of cellular physiology in a dynamic cellular microenvironment [41,42,43,44]. The additional recent trend to combine microfluidics with non-invasive biosensors has created new opportunities to obtain time-resolved and dynamic information on the health status of *in vitro* cell cultures [45,46,47,48].

In the present study, we have developed a microfluidic cell culture system containing integrated impedance microsensors to continuously and non-invasively monitor the regenerative capacity of human lung adenocarcinoma cells following dynamic administration of silica nanoparticles. The main advantage of our microfluidic regeneration assay is the paradigm shift from conventional static to perfused nanoparticle administration under multiple flow conditions, which is accompanied by a severe impact on the nanomaterial-cell interaction, thus the cytotoxic potential of nanomaterials. Overall, these results demonstrate that in addition to the physical (e.g., size), chemical (e.g., surface coating) and biological (e.g., toxicity) characteristics of the nanoparticle, cellular nanoparticle uptake and toxicity is also governed by applied fluidic exposure scenarios. Furthermore, impedance time traces recorded with integrated biosensors revealed that only the ability to continuously assess the regeneration behavior provides adequate information on the dynamic cellular behavior, especially for concentrations below the cytotoxicity threshold. The presented results indicate that apart from the concentration of nanoparticles, as well as media composition, also the strategy of nanoparticle administration severely impacts cell viability and regeneration, thus the bioactivity of nanomaterials. Consequently, in addition to perfused administration scenarios, continuous monitoring of cellular responses before, during and after nanoparticle exposure needs to be considered for future *in vitro* nanotoxicology assays.

## Supplementary Files

Supplementary File 1

## References

[1] Moghimi S.M., Hunter A.C., Murray J.C. (2005). Nanomedicine: Current status and future prospects. FASEB.

[2] Freitas R.A. (1999). Nanomedicine, Volume I: Basic Capabilities.

[3] Poole C.P., Owens F.J. (2003). Introduction to Nanotechnology.

[4] Santamaria A., Reineke J. (2012). Historical overview of nanotechnology and nanotoxicology. Nanotoxicity: Methods and Protocols.

[5] Xu Z.P., Zeng Q.H., Lu G.Q., Yu A.B. (2006). Inorganic nanoparticles as carriers for efficient cellular delivery. Chem. Eng. Sci..

[6] Maeda M., Kuroda C.S., Shimura T., Tada M., Abe M., Yamamuro S., Sumiyama K., Handa H. (2006). Magnetic carriers of iron nanoparticles coated with a functional polymer for high throughput bioscreening. J. Appl. Phys..

[7] Hilger I., Hergt R., Kaiser W.A. (2005). Use of magnetic nanoparticle heating in the treatment of breast cancer. IEEE Proc. Nanobiotechnol..

[8] Manke A., Wang L., Rojanasakul Y. (2013). Mechanisms of nanoparticle-induced oxidative stress and toxicity. BioMed Res. Int..

[9] AshaRani P.V., Low Kah Mun G., Hande M.P., Valiyaveettil S. (2009). Cytotoxicity and genotoxicity of silver nanoparticles in human cells. ACS Nano.

[10] Lunov O., Syrovets T., Rocker C., Tron K., Nienhaus G.U., Rasche V., Mailander V., Landfester K., Simmet T. (2010). Lysosomal degradation of the carboxydextran shell of coated superparamagnetic iron oxide nanoparticles and the fate of professional phagocytes. Biomaterials.

[11] Johnston H.J., Hutchison G., Christensen F.M., Peters S., Hankin S., Stone V. (2010). A review of the *in vivo* and *in vitro* toxicity of silver and gold particulates: Particle attributes and biological mechanisms responsible for the observed toxicity. Crit. Rev. Toxicol..

[12] Ju-Nam Y., Lead J.R. (2008). Manufactured nanoparticles: An overview of their chemistry, interactions and potential environmental implications. Sci. Total Environ..

[13] Li N., Xia T., Nel A.E. (2008). The role of oxidative stress in ambient particulate matter-induced lung diseases and its implications in the toxicity of engineered nanoparticles. Free Radic. Biol. Med..

[14] Stone V., Johnston H., Clift M.J. (2007). Air pollution, ultrafine and nanoparticle toxicology: Cellular and molecular interactions. IEEE Trans. Nanobiosci..

[15] Nystrom A.M., Fadeel B. (2012). Safety assessment of nanomaterials: Implications for nanomedicine. J. Controll. Release.

[16] Baun A., Hansen S.F. (2008). Environmental challenges for nanomedicine. Nanomedicine.

[17] Bawa R. (2011). Regulating nanomedicine—Can the fda handle it?. Curr. Drug Deliv..

[18] Rahman M., Ahmad M.Z., Kazmi I., Akhter S., Afzal M., Gupta G., Sinha V.R. (2012). Emergence of nanomedicine as cancer targeted magic bullets: Recent development and need to address the toxicity apprehension. Curr. Drug Discov. Technol..

[19] Kramer N., Walzl A., Unger C., Rosner M., Krupitza G., Hengstschlager M., Dolznig H. (2013). *In vitro* cell migration and invasion assays. Mutat. Res..

[20] Richter L., Charwat V., Jungreuthmayer C., Bellutti F., Brueckl H., Ertl P. (2011). Monitoring cellular stress responses to nanoparticles using a lab-on-a-chip. Lab Chip.

[21] Park M.S., Yoon T.H. (2014). Effects of ag nanoparticle flow rates on the progress of the cell cycle under continuously flowing “dynamic” exposure conditions. Bull. Korean Chem. Soc..

[22] Mahto S.K., Yoon T.H., Rhee S.W. (2010). A new perspective on *in vitro* assessment method for evaluating quantum dot toxicity by using microfluidics technology. Biomicrofluidics.

[23] Sticker D., Rothbauer M., Charwat V., Steinkühler J., Bethge O., Bertagnolli E., Wanzenboeck H.D., Ertl P. (2015). Zirconium dioxide nanolayer passivated impedimetric sensors for cell-based assays. Sens. Actuat. B: Chem..

[24] Docter D., Bantz C., Westmeier D., Galla H.J., Wang Q., Kirkpatrick J.C., Nielsen P., Maskos M., Stauber R.H. (2014). The protein corona protects against size- and dose-dependent toxicity of amorphous silica nanoparticles. Beilstein J. Nanotechnol..

[25] Docter D., Strieth S., Westmeier D., Hayden O., Gao M., Knauer S.K., Stauber R.H. (2015). No king without a crown—Impact of the nanomaterial-protein corona on nanobiomedicine. Nanomedicine.

[26] Docter D., Westmeier D., Markiewicz M., Stolte S., Knauer S.K., Stauber R.H. (2015). The nanoparticle biomolecule corona: Lessons learned—Challenge accepted?. Chem. Soc. Rev..

[27] Thakur M., Mergel K., Weng A., Frech S., Gilabert-Oriol R., Bachran D., Melzig M.F., Fuchs H. (2012). Real time monitoring of the cell viability during treatment with tumor-targeted toxins and saponins using impedance measurement. Biosens. Bioelectron..

[28] Liu Q.J., Yu J.J., Xiao L., Tang J.C.O., Zhang Y., Wang P., Yang M. (2009). Impedance studies of bio-behavior and chemosensitivity of cancer cells by micro-electrode arrays. Biosens. Bioelectron..

[29] Yu J., Liu Z., Yang M., Mak A. (2009). Nanoporous membrane-based cell chip for the study of anti-cancer drug effect of retinoic acid with impedance spectroscopy. Talanta.

[30] Qiao G.F., Wang W., Duan W., Zheng F., Sinclair A.J., Chatwin C.R. (2012). Bioimpedance analysis for the characterization of breast cancer cells in suspension. IEEE Trans. Biomed. Eng..

[31] Cho Y., Kim H.S., Frazier A.B., Chen Z.G., Shin D.M., Han A. (2009). Whole-cell impedance analysis for highly and poorly metastatic cancer cells. J. Microelectromech. Syst..

[32] Srinivasaraghavan V., Strobl J., Agah M. (2012). Bioimpedance rise in response to histone deacetylase inhibitor is a marker of mammary cancer cells within a mixed culture of normal breast cells. Lab Chip.

[33] Samuel S.P., Jain N., O’Dowd F., Paul T., Kashanin D., Gerard V.A., Gun’ko Y.K., Prina-Mello A., Volkov Y. (2012). Multifactorial determinants that govern nanoparticle uptake by human endothelial cells under flow. Nanomedicine.

[34] Freese C., Schreiner D., Anspach L., Bantz C., Maskos M., Unger R.E., Kirkpatrick C.J. (2014). *In vitro* investigation of silica nanoparticle uptake into human endothelial cells under physiological cyclic stretch. Part. Fibre Toxicol..

[35] Tenzer S., Docter D., Kuharev J., Musyanovych A., Fetz V., Hecht R., Schlenk F., Fischer D., Kiouptsi K., Reinhardt C. (2013). Rapid formation of plasma protein corona critically affects nanoparticle pathophysiology. Nat. Nanotechnol..

[36] Charwat V., Joksch M., Sticker D., Purtscher M., Rothbauer M., Ertl P. (2014). Monitoring cellular stress responses using integrated high-frequency impedance spectroscopy and time-resolved elisa. Analyst.

[37] Charwat V., Rothbauer M., Tedde S.F., Hayden O., Bosch J.J., Muellner P., Hainberger R., Ertl P. (2013). Monitoring dynamic interactions of tumor cells with tissue and immune cells in a lab-on-a-chip. Anal. Chem..

[38] Zhang X., Li F., Nordin A.N., Tarbell J., Voiculescu I. (2015). Toxicity studies using mammalian cells and impedance spectroscopy method. Sens. Bio-Sens. Res..

[39] Park S., Choi J., Kim S. (2015). Measurement of cell-substrate impedance and characterization of cancer cell growth kinetics with mathematical model. Int. J. Precis. Eng. Manuf..

[40] Koppenhofer D., Susloparova A., Docter D., Stauber R.H., Ingebrandt S. (2013). Monitoring nanoparticle induced cell death in h441 cells using field-effect transistors. Biosens. Bioelectron..

[41] Chiu D.T., Jeon N.L., Huang S., Kane R.S., Wargo C.J., Choi I.S., Ingber D.E., Whitesides G.M. (2000). Patterned deposition of cells and proteins onto surfaces by using three-dimensional microfluidic systems. Proc. Natl. Acad. Sci. USA.

[42] Takayama S., Ostuni E., Qian X.P., McDonald J.C., Jiang X.Y., LeDuc P., Wu M.H., Ingber D.E., Whitesides G.M. (2001). Topographical micropatterning of poly(dimethylsiloxane) using laminar flows of liquids in capillaries. Adv. Mater..

[43] Bransky A., Korin N., Levenberg S. (2008). Experimental and theoretical study of selective protein deposition using focused micro laminar flows. Biomed. Microdevices.

[44] Wang L., Lei L., Ni X.F., Shi J., Chen Y. (2009). Patterning bio-molecules for cell attachment at single cell levels in pdms microfluidic chips. Microelectron. Eng..

[45] Fang Y. (2011). Label-free biosensors for cell biology. Int. J. Electrochem..

[46] Mahto S.K., Charwat V., Ertl P., Rothen-Rutishauser B., Rhee S.W., Sznitman J. (2015). Microfluidic platforms for advanced risk assessments of nanomaterials. Nanotoxicology.

[47] Rothbauer M., Wartmann D., Charwat V., Ertl P. (2015). Recent advances and future applications of microfluidic live-cell microarrays. Biotechnol. Adv..

[48] Wartmann D., Rothbauer M., Kuten O., Barresi C., Visus C., Felzmann T., Ertl P. (2015). Automated, miniaturized and integrated quality control-on-chip (qc-on-a-chip) for advanced cell therapy applications. Front. Mater..

