# Supplementary material for: Microfluidic Impedimetric Cell Regeneration Assay to Monitor the Enhanced Cytotoxic Effect of Nanomaterial Perfusion
Source: Biosensors (Basel). 2015 Nov 27;5(4):736–49. doi: 10.3390/bios5040736 (PMC4697142; doi:10.3390/bios5040736)
Supplement: Supplementary File 1 [file biosensors-05-00736-s001.pdf]

OPEN ACCESS
biosensors
ISSN 2079-6374
www.mdpi.com/journal/biosensors/

Supplementary Information

Microfluidic Impedimetric Cell Regeneration Assay to Monitor the Enhanced Cytotoxic Effect of Nanomaterial Perfusion. *Biosensors* 2015, *4*, 736-749

Mario Rothbauer [1], Irene Praisler [1], Dominic Docter [2], Roland H. Stauber[2] and Peter Ertl [1,*]

[1] BioSensor Technologies, AIT Austrian Institute of Technology GmbH, 1190 Vienna, Austria; E-Mails: mario.rothbauer@gmail.com (M.R.); ipraisler@gmail.com (I.P.)
[2] Molecular and Cellular Oncology, ENT/University Medical Center Mainz, 55116 Mainz, Germany; E-Mails: docter@uni-mainz.de (D.D.); rstauber@uni-mainz.de (R.H.S.)

***** Author to whom correspondence should be addressed; E-Mail: peter.ertl@ait.ac.at; Tel.: +43-50550-4469; Fax: +43-50550-4450.

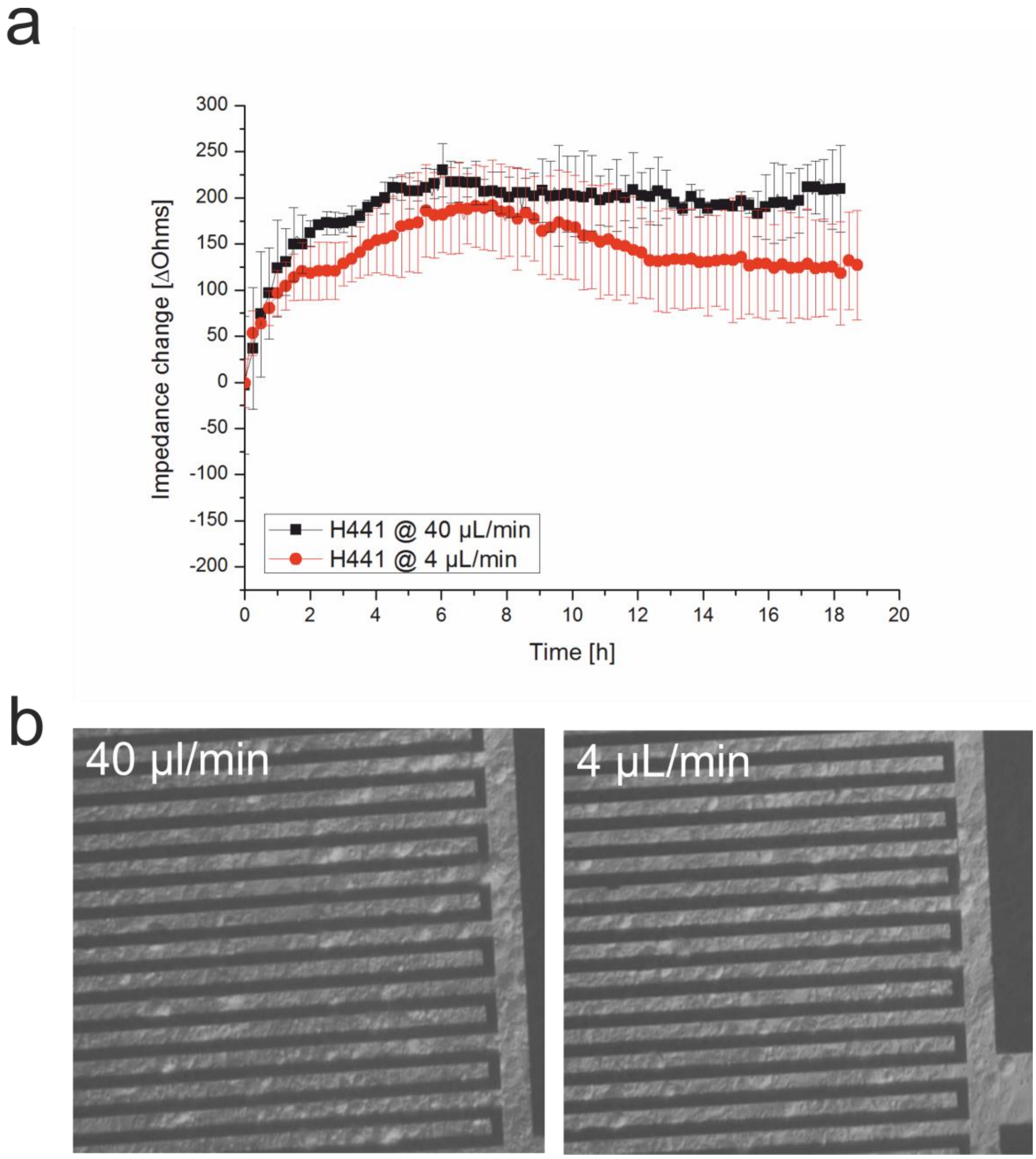

Figure S1.

© 2015 by the authors; licensee MDPI, Basel, Switzerland. This article is an open access article distributed under the terms and conditions of the Creative Commons Attribution license (http://creativecommons.org/licenses/by/4.0/).
